# Managing variability in decision making in swine growing-finishing units

**DOI:** 10.1186/s13620-015-0048-z

**Published:** 2015-09-01

**Authors:** Piero da Silva Agostini, Edgar Garcia Manzanilla, Carlos de Blas, Alan G. Fahey, Caio Abercio da Silva, Josep Gasa

**Affiliations:** Grup de Nutrició, Maneig i Benestar Animal, Department de Ciència Animal i dels Aliments, Universitat Autònoma de Barcelona, Bellaterra, 08193 Spain; Pig Development Department, Animal and Grassland Research and Innovation Centre, Teagasc, Moorepark, Fermoy, Co. Cork, Ireland; Departamento de Producción Animal, Escuela Técnica de Ingenieros Agrónomos, Universidad Politécnica de Madrid, Madrid, 28040 Spain; School of Agriculture and Food Science, University College Dublin, Dublin 4, Belfield Ireland; Departamento de Zootecnia, Universidade Estadual de Londrina, Londrina, 86051-970 Brazil

**Keywords:** Facilities, Finishing pig, Management, Mortality, Regression

## Abstract

**Background:**

Analysis of data collected from pig farms may be useful to understand factors affecting pig health and productive performance. However, obtaining these data and drawing conclusions from them can be done at different levels and presents several challenges. In the present study, information from 688 batches of growing-finishing (GF) pigs (average initial and final body weight of 19.1 and 108.5 kg respectively) from 404 GF farms integrated in 7 companies was obtained between July 2008 and July 2010 in Spain by survey. Management and facility factors associated with feed conversion ratio (FCR) and mortality were studied by multiple linear regression analysis in each single company (A to G) and in an overall database (OD). Factors studied were geographic location of the farm, trimester the pigs entered the farm, breed of sire and sex segregation in pens (BREGENSEG), use of circovirus vaccine, number of origins the pigs were obtained from, age of the farm, percentage of slatted floor, type of feeder, drinker and ventilation, number of phases and form of feed, antibiotic administration system, water source, and number and initial weight of pigs.

**Results:**

In two or more companies studied and/or in OD, the trimester when pigs were placed in the farm, BREGENSEG, number of origins of the pigs, age of the farm and initial body weight were factors associated with FCR. Regarding mortality, trimester of placement, number of origins of the pigs, water source in the farm, number of pigs placed and the initial body weight were relevant factors. Age of the farm, antibiotic administration system, and water source were only provided by some of the studied companies and were not included in the OD model, however, when analyzed in particular companies these three variables had an important effect and may be variables of interest in companies that do not record them.

**Conclusions:**

Analysing data collected from farms at different levels helps better understand factors associated with productive performance of pig herds. Out of the studied factors trimester of placement and number of origins of the pigs were the most relevant factors associated with FCR and mortality.

## Background

The grow-finishing (GF) phase, defined as the period between the exit of nursery (when the animal weights around 20 kg) and slaughter house (>105 kg), is considered the most expensive component of pig production accounting for approximately 69 % of the costs pork meat production in Spain [1] as in most countries. In the last 15 years studies have been conducted on the association between different management factors and suboptimal health [2–4] or productive performance [5–7] during the GF phase through the use of prediction models. These studies used data from commercial farms or batches of pigs belonging to a single pig producing company. However, in recent years companies have tended to homogenize the management of their herds through increased uniformity of their facilities (feeders, drinkers, floor types, ventilation systems, or other production conditions) and health management routines (vaccination, or antibiotic treatments). There is little information in the literature about the variability among companies or about the effects of management and facilities on performance traits using data from different companies. Variability among companies may be as informative as variability within company and integrating information from different companies may be useful in aideing management decisions. There are different approaches in order to integrate this information and using more than one approach simultaneously may provide us with extra valuable information. Therefore, the objective of this study was to collect data from pig producing companies representative of the production in Spain and determine the effect of animal management, temporal and geographical context and farm facilities on feed conversion ratio (FCR) and mortality rate (MORT) in GF pigs belonging to different companies using two different linear regression approaches.

## Methods

### Sampling information and variables collected in the study

A cross-sectional study was developed including information from a total of 688 batches of pigs from 404 GF farms (one to three batches per farm) integrated in seven Spanish companies (abbreviated as “Com” from “A” to “G”) with a combined total of 1,040,116 pigs. The study protocol was discussed with the 25 largest growing-finishing pig companies in Spain. Seven of them were selected to participate in this study based on the availability of data and being representative of the main geographic areas producing pigs in Spain. Companies were asked to provide data regarding performance responses from a minimum of 30 batches. The companies were asked to provide batches from different trimesters but no other criteria were applied in the selection to obtain representative data of the reality of each company. Information about the farms was collected by farm survey between July 2008 and July 2010 using a questionnaire model prepared by the research team in collaboration with the field veterinarians participating in the project.

Variables that had been shown to affect production of GF pigs were selected from literature [5–9]. The categorical and continuous variables used in each company are described in Tables 1 and 2. In all batches, the pigs remained in the GF unit from completion of the nursery phase until slaughter and were managed in an all-in all-out system. Pig companies were located in the three Spanish regions where most pig meat is produced; Cataluña, Aragón, and Castilla y León.

**Table 1:** Characterization of the variables recorded from 688 batches in 404 farms belonging to seven integrated pigs companies

		Company						
		A	B	C	D	E	F	G
NBATCHES		207	97	99	99	74	33	79
REGION	Catauña	48.3 %	-	47.5 %	-	-	100 %	-
	Aragón	51.7 %	-	36.4 %	100 %	100 %	-	10.1 %
	Castilla y León	-	100 %	-	-	-	-	82.3 %
	Valencia	-	-	16.1 %	-	-	-	-
	Other	-	-	-	-	-	-	7.6 %
TRIMESTER	Jan-Feb-Mar	-	26.8 %	-	24.2 %	25.7 %	-	27.8 %
	Apr-May-Jun	34.8 %	24.7 %	56.6 %	25.3 %	21.6 %	51.6 %	12.7 %
	Jul-Aug-Sep	-	20.6 %	-	31.3 %	31.1 %	-	27.8 %
	Oct-Nov-Dec	65.2 %	27.8 %	43.4 %	19.2 %	21.6 %	48.4 %	31.7 %
PIGFAT	Industrial 95–110 kg	100 %	100 %	100 %	100 %	100 %	100 %	-
	Heavy > 110 kg	-	-	-	-	-	-	100 %
SEX	Male and female	100 %	64.9 %	59.6 %	100 %	100 %	100 %	-
	Barrow and female	-	35.1 %	40.4 %	-	-	-	100 %
SPLITSEX	Split-sex	100 %	-	-	100 %	-	100 %	-
	Mixed-sex	-	100 %	100 %	-	100 %	-	100 %
BREED	Pietrain	100 %	64.9 %	36.4 %	100 %	100 %	100 %	-
	White	-	35.1 %	45.4 %	-	-	-	100 %
	Duroc	-	-	18.2 %	-	-	-	-
CIRCOVAC	No	34.3 %	100 %	80.8 %	100 %	100 %	100 %	100 %
	Yes	65.7 %	-	19.2 %	-	-	-	-
ORIGIN	One	39.6 %	49.5 %	22.2 %	100 %	100 %	100 %	40.5 %
	More than one	0.4 %	50.5 %	77.8 %	-	-	-	59.5 %
AGEBARN	<10 years	Not available	14.4 %	-	28.3 %	39.2 %	29.0 %	27.8 %
	10 to 30 years		41.2 %	100 %	71.7 %	60.8 %	71.0 %	72.3 %
	>30 years		44.3 %	-	-	-	-	-
PIGPEN	<10 pigs	-	54.6 %	24.1 %	-	-	-	5.1 %
	10 to 20 pigs	100 %	45.4 %	75.9 %	100 %	100 %	100 %	94.9 %
FLOOR	<50 % slatted	-	29.9 %	45.7 %	37.4 %	100 %	48.3 %	12.7 %
	≥50 % slatted	100 %	70.1 %	54.3 %	62.6 %	-	51.7 %	87.3 %
FEEDER	Multiple-space	-	58.8 %	100 %	-	6.8 %	-	16.9 %
	Single-space	100 %	19.6 %	-	100 %	-	19.4 %	-
	Single-space + drinker	-	21.6 %	-	-	93.2 %	80.6 %	83.1 %
DRINKER	Nipple	100 %	40.2 %	100 %	100 %	100 %	45.4 %	100 %
	Bowl	-	36.1 %	-	-	-	54.6 %	-
	Without drinker^a^	-	23.7 %	-	-	-	-	-
VENT	Manual	-	71.1 %	100 %	-	75.7 %	9.7 %	-
	Automatic	100 %	28.9 %	-	100 %	24.3 %	90.3 %	100 %
FPHASE	Three	100 %	100 %	-	100 %	100 %	100 %	-
	Four	-	-	100 %	-	-	-	100 %
FFORM	Pellet	100 %	100 %	100 %	100 %	100 %	100 %	-
	Meal	-	-	-	-	-	-	100 %
PATHATB	Feed	-	50.5 %	-	100 %	-	Not available	-
	Water + feed	-	49.5 %	-	-	100 %		-
	Feed + injection	-	-	-	-	-		100 %
	Water + feed + injection	100 %	-	100 %	-	-		-
WATERSOU	Well	9.9 %	85.6 %	Not available	-	-	-	100 %
	River	53.5 %	-		-	-	-	-
	Public water	28.2 %	14.4 %		100 %	40.5 %	100 %	-
	Others	8.4 %	-		-	59.5 %	-	-
NPP	<800 pigs	19.8 %	54.6 %	15.2 %	8.1 %	-	35.5 %	11.4 %
	800 to 2000 pigs	58.0 %	45.4 %	57.6 %	43.4 %	58.1 %	54.8 %	60.8 %
	>2000 pigs	22.2 %	-	27.2 %	48.5 %	41.9 %	9.7 %	27.8 %

NBATCHES - number of batches; REGION - region from Spain; TRIMESTER - trimester of placement; PIGFAT - type of pigs fattened; SEX - sex present at batches; SPLITSEX - sex segregation in pens; BREED - breed of the pig-sire; CIRCOVAC - circovirus vaccine; ORIGIN - number of pig origins; AGEBARN - age of the herds; PIGPEN - number of pigs per pen; FLOOR - percentage of slatted floor; FEEDER - type of feeder; DRINKER - type of drinker; VENT - ventilation control system; FPHASE - number of feed phases; FFORM - feed form; PATHATB - routes used to perform treatments; WATERSOU - water source in the herd; NPP - number of pigs placed

^a^Animals had access to water through the drinkers coupled in the feeders

**Table 2:** Number of pigs per batch, initial and final body weight, feed conversion ratio and mortality rate^a^

				Production parameters							
		Number of pigs		IBW (kg)		FBW (kg)		FCR (kg/kg)		sqrtMORT	
Company	N	Mean	SD	Mean	SD	Mean	SD	Mean	SD	Mean	SD
A	207	1433	750	19.2	1.6	104.8	4.5	2.70	0.13	2.01	0.60
B	97	802	433	21.0	1.4	106.7	4.8	2.83	0.17	1.68	0.51
C	99	1564	918	18.8	1.9	106.4	3.4	2.89	0.22	2.61	0.64
D	99	2125	1309	16.5	1.8	108.2	2.6	2.70	0.09	1.62	0.40
E	74	1763	773	17.4	1.8	107.1	3.1	2.62	0.10	1.84	0.44
F	33	1218	689	20.1	5.4	106.9	3.6	2.62	0.09	1.82	0.47
G	79	1673	1041	20.7	2.0	119.7	3.5	2.90	0.11	1.90	0.37
OD^b^	607	1496	941	18.8	2.8	106.3	4.1	2.74	0.17	1.96	0.63

^a^Data from 688 batches in 404 growth-finishing farms belonging to seven integrated pigs companies. SD: standard deviation

^b^OD - database of companies A to F. Initial body weight (IBW) refers to pig live weight when entering the GF unit. Final body weight (FBW) was the live weight recorded in the GF unit prior to transportation to the slaughter facility. Feed conversion ratio (FCR) was obtained dividing the total feed delivered to each batch (kg) by the difference between the total kilograms of pigs sent to slaughter and the total kilograms of pigs that entered a GF batch. Finally, square root of the mortality rate (sqrtMORT) was calculated as the square root of the number of nursery pigs that entered the GF unit minus the number of pigs transported for slaughter divided by the number of pigs that entered the GF unit

### Data processing and analyses

Data were analyzed using SAS 9.2 (SAS, Cary, NC, USA). Batch was considered the experimental unit for all analyses. Descriptive statistics were performed for continuous and categorical variables and the distributions of their residuals were examined. Mortality rate data were square root transformed (sqrtMORT) in order to achieve the assumptions of linear regression. Feed Conversion Ratio and sqrtMORT were the dependent variables and all categorical variables (Table 1) and initial body weight (IBW) were considered as predictor variables, with IBW remaining in all final statisitcal models regardless of the *P-*value. Dependent variables were analyzed using linear mixed effects regression models. Variance components were estimated using REML.

The effects of independent variables on the dependent variables were studied using two approaches: 1) data were analysed separately for each of the seven companies, including company specific models that only contained factors that had variability for the company, and 2) data were analyzed combining data from all companies at the same time in one model using an overall database (OD). One of the companies was not included in the OD because it fattened pigs to higher FBW compared to th other companies (119.70 vs. 106.61 kg) and this kind of production presents very particular conditions. Variables not provided by one or more of the companies were not taken into account in the OD model in order to avoid reductions in sample size. Farm was not considered as a random effect because most of the farms contributed one batch to the database. Company was included initially as a random factor but it was not significant (*P* > 0.10) and then it was excluded from the models.

Initially, a univariate regression model was used where each predictor variable was included as a single fixed factor to predict. Variables that had *P* < 0.25 in the univariate analysis were selected for use in the multivariable analysis [10] in the MIXED procedure of SAS. Before entering the variables into a multivariable model, bivariate Pearson’s and Spearman’s correlations were performed among independent variables in order to avoid multicollinearity problems between the continuous variables and confounding problems between the categorical variables. There were no high correlations between any of the continuous variables selected (r < 0.60) and all were included in the multivariable models. However, breed of sire, sex, and sex segregation in batches showed a relationship being used only in particular combinations. Thus, these variables were grouped as a single combined variable (BREGENSEG). The model was built using a manual stepwise regression model procedure; all factors with a *P* < 0.10 were retained in the final model. Finally, all two-way interactions between significant variables in the multivariable model were tested and included if *P* < 0.05. After fitting the conditional models for each dependent variable, both normality and homoscedasticity of the residuals were evaluated.

## Results

A total of 688 batches housed in 404 GF farms integrated in seven companies (average of 98 batches and 148,588 pigs per company) were analyzed separately by company as well as grouped (n = 607 batches from 363 herds). Mean and SD for IBW and FBW for OD data were 18.8 ± 2.48 kg and 106.3 ± 4.12 kg respectively. Details for each company are shown in Table 2.

### Feed conversion ratio

The mean and SD for FCR of the OD was 2.74 ± 0.17 kg/kg (with 25^th^ and 75^th^ percentiles of 2.62 and 2.84 kg/kg respectively) and the multivariable model for OD had an r^2^ = 0.27. Means and SD of FCR found for each company are shown in Table 2. Multivariable regression analysis (Table 3) indicated that in ComC the FCR was 0.09 poorer on average when batches were located in Cataluña compared to Aragón (*P* < 0.10). The FCR was 0.07 better when pigs were placed between April-June at ComC (*P* < 0.10) and 0.09 when they were placed between January-March at ComE (*P* < 0.01) in comparison to those placed in October-December. Moreover, the effect of the trimester of placement on FCR varied depending on: 1) circovirus vaccine at ComA; 2) BREGENSEG at ComB, and 3) initial body weight at OD. The use of White and Duroc sired pigs in the presence of barrow and mixed-sex batches had 0.15 (*P* < 0.01) and 0.13 (*P* < 0.10) poorer FCR respectively compared to those batches that had a Pietrain sire-pig, males, and mixed-sex at ComC. Furthermore, the influence of the BREGENSEG on FCR was modified by the season of placement (ComB) and initial body weight (OD). The effect of the use of a vaccine against circovirus disease on FCR depended on the season of placement at ComA. Batches containing pigs from a unique origin had 0.10, 0.04, and 0.05 better FCR at ComC (*P* < 0.10), ComG (*P* < 0.10), and OD (*P* < 0.01) respectively than when they came from multiple origins. The ComF had a poorer FCR (0.07) in batches belonging to farms which were between 10 and 30 years old than barns that were less than 10 years old (*P* < 0.05). Moreover, at ComD the effect of herd age on FCR was modified when IBW varied. An improved FCR (0.04) was found in batches from farms in ComE (*P* < 0.10) which did not obtain the water from a public supply. For OD, there was an improvement in FCR by 0.08 and 0.06 when pigs were fed in a single-space feeder with an incorporated drinker compared to both multi and single-space feeder without any incorporated drinker respectively (*P* < 0.001). The FCR was 0.05 poorer in batches containing between 800 and 2000 pigs at ComA (*P* < 0.05) and about 0.16 poorer in batches containing more than 2000 pigs at ComC (*P* < 0.05) in comparison to batches containing less than 800 pigs. Finally, for every 1 kg increase in IBW, FCR was 0.020, 0.031, 0.025 and 0.025 poorer in ComA*,* ComD, ComG and OD respectively (*P* < 0.001).

**Table 3:** Parameter estimates (standard error in parenthesis) for the feed conversion ratio (FCR) in each company studied^a^

Variable	Companies							
	A	B	C	D	E	F	G	OD^b^
Intercept	2.26 (0.10)^***^	3.09 (0.21)^***^	2.78 (0.25)^***^	2.16 (0.13)^***^	2.60 (0.12)^***^	2.54 (0.06)^***^	2.41 (0.12)^***^	2.26 (0.11)^***^
Autonomous community								
Cataluña	S	U	0.09 (0.05)^****^	U	U	U	S	S
Castilla y León	S	U	A	U	U	U	S	S
Valencia	S	U	- 0.06 (0.06)	U	U	U	S	S
Other	S	U	A	U	U	U	S	S
Aragón	S	U	–	U	U	U	S	S
Trimester of placement								
Jan-Feb-Mar	A	−0.24 (0.05)^***^	A	S	−0.09 (0.03)^**^	S	S	−0.03 (0.12)
Apr-May-Jun	−0.03 (0.02)^*^	−0.33 (0.04)^***^	−0.07 (0.04)^****^	S	−0.02 (0.03)	S	S	0.15 (0.09)
Jul-Aug-Sep	A	−0.31 (0.05)^***^	A	S	−0.01 (0.03)	S	S	0.30 (0.10)^**^
Oct-Nov-Dec	–	–	–	S	–	S	S	–
BREGENSEG^c^								
Pietrain – Male – Mixed-sex	U	–	–	U	U	U	U	–
Pietrain – Male – Split-sex	U	A	A	U	U	U	U	0.24 (0.10)^*^
White – Male – Mixed-sex	U	A	0.04 (0.06)	U	U	U	U	0.57 (0.34)^****^
White – Barrow – Mixed-sex	U	−0.05 (0.05)^****^	0.15 (0.06)^**^	U	U	U	U	0.53 (0.23)^*^
Duroc – Barrow – Mixed-sex	U	A	0.13 (0.07)^****^	U	U	U	U	0.83 (0.26)^**^
Circovirus vaccine								
No	0.12 (0.02)^***^	U	S	U	U	U	U	S
Yes	–	U	S	U	U	U	U	S
Number of pig origins								
One origin	S	S	−0.10 (0.06)^****^	U	U	U	−0.04 (0.02)^****^	−0.05 (0.01)^**^
More than one origin	S	S	–	U	U	U	–	–
Herd age								
<10 years	A	S	U	–	S	–	S	A
10 to 30 years	A	S	U	0.50 (0.16)^**^	S	0.07 (0.03)^*^	S	A
>30 years	A	S	U	A	S	A	S	A
Type of feeder								
Multi-space	U	S	U	U	S	S	S	0.07 (0.02)^***^
Single-space	U	S	U	U	S	S	S	0.06 (0.02)^***^
Single-space with drinker	U	S	U	U	S	S	S	–
Routes utilized to perform treatments								
Feed	U	S	U	U	U	A	U	A
Feed + Water	U	S	U	U	U	A	U	A
Feed + Water + Injection	U	S	U	U	U	A	U	A
Water source in the farm								
Well	S	S	A	U	A	U	U	A
River	S	S	A	U	A	U	U	A
Other	S	S	A	U	−0.04 (0.02)^****^	U	U	A
Public supply	S	S	A	U	–	U	U	A
Number of animals placed								
<800 pigs	–	S	–	S	A	S	S	S
800-2000 pigs	−0.04 (0.02)^*^	S	0.09 (0.06)	S	S	S	S	S
>2000 pigs	−0.01 (0.02)	S	0.16 (0.07)^*^	S	S	S	S	S
Initial body weight	0.02 (0.005)^***^	−0.004 (0.009)	−0.001 (0.01)	0.03 (0.008)^***^	0.005 (0.01)	0.001 (0.003)	0.02 (0.005)^***^	0.02 (0.01)^***^
Trimester * Circovirus vaccine								
Apr-May-Jun / No vaccinated	0.14 (0.04)^***^	U	U	U	U	U	U	U
/ Vaccinated	–	U	U	U	U	U	U	U
Oct-Nov-Dec / No vaccinated	–	U	U	U	U	U	U	U
/ Vaccinated	–	U	U	U	U	U	U	U
Initial body weight * Herd age								
Initial body weight / < 10 years	U	U	U	–	U	U	U	U
/ 10 to 30 years	U	U	U	−0.03 (0.01)^**^	U	U	U	U
Initial body weight * Trimester								
Initial body weight / Jan-Feb-Mar	U	U	U	U	U	U	U	−0.0005 (0.006)
/ Apr-May-Jun	U	U	U	U	U	U	U	−0.01 (0.005)^*^
/ Jul-Aug-Sep	U	U	U	U	U	U	U	−0.02 (0.005)^**^
/ Oct-Nov-Dec	U	U	U	U	U	U	U	–
Trimester of placement * BREGENSEG								
Jan-Feb-Mar / Pietrain – Male – Mixed-sex	U	–	U	U	U	U	U	U
/ White – Barrow – Mixed-sex	U	0.23 (0.07)^***^	U	U	U	U	U	U
Apr-May-Jun / Pietrain – Male – Mixed-sex	U	–	U	U	U	U	U	U
/ White – Barrow – Mixed-sex	U	0.20 (0.09)^*^	U	U	U	U	U	U
Jul-Aug-Sep / Pietrain – Male – Mixed-sex	U	–	U	U	U	U	U	U
/ White – Barrow – Mixed-sex	U	0.28 (0.08)^***^	U	U	U	U	U	U
Oct-Nov-Dec / Pietrain – Male – Mixed-sex	U	–	U	U	U	U	U	U
/ White – Barrow – Mixed-sex	U	–	U	U	U	U	U	U
Initial BW * BREGENSEG								
Initial BW / Pietrain - Male - Mixed-sex	U	U	U	U	U	U	U	–
/ Pietrain - Male - Split-sex	U	U	U	U	U	U	U	−0.01 (0.005)^**^
/ White - Male - Mixed-sex	U	U	U	U	U	U	U	−0.02 (0.02)
/ White - Barrow - Mixed-sex	U	U	U	U	U	U	U	−0.02 (0.01)^****^
/ Duroc - Barrow - Maxed-sex	U	U	U	U	U	U	U	−0.03 (0.01)^*^

S - Variable was not selected for the final model; U - Variable non-used in the model because it did not present variability; A - Variable or level not available

(−) Reference level for a factor included in the multiple regression models

^*^ *P* < 0.05; ^**^ *P* < 0.01; ^***^ *P* < 0.001; ^****^ *P* < 0.10

^a^Data from 688 batches for companies A to F and 607 batches for OD. Variables not significant with respect to FCR in all companies studied and in OD were not described at the table

^b^OD - overall database. Data from ¨company G¨ was not included

^c^BREGENSEG - This variable combined ¨breed of the pig-sire¨, ¨sex present in batches¨ and ¨sex segregation in batches¨

### Square root of mortality rate

Mean and SD for sqrtMORT for the OD was 1.96 ± 1.49 (with 25^th^ and 75^th^ percentiles of 1.54 and 2.39 kg/kg respectively) and the multivariable model for OD had an r^2^ = 0.20. Means and SD for sqrtMORT found for each company are shown in Table 2. Multivariable regression analysis (Table 4) indicated that in ComC, batches in farms located in Cataluña tended to have a higher sqrtMORT of 0.25 (*P* < 0.10) compared to farms from Aragón. A reduction in sqrtMORT was observed when pigs were placed in farms in warm seasons. In particular, when compared to pigs placed between October-December, pigs in ComB that entered farms between April-June and July-September decreased sqrtMORT by 0.45 (*P* < 0.001) and 0.43 (*P* < 0.01) respectively. Pigs placed from April to June reduced sqrtMORT by 0.25 and 0.35 in ComC and ComF respectively (*P* < 0.05); batches that entered farms from January to March had an increase of 0.30 in sqrtMORT (*P* < 0.05) in ComE. There was also a reduction in sqrtMORT by 0.26 (*P* < 0.001) and 0.28 (*P* < 0.001) when pigs were placed between April-June and July-September respectively in the OD. Finally, the effect of season on sqrtMORT depended on the use of a circovirus vaccine at ComA. Batches that contained pigs from a unique origin had a decrease by 0.16 (*P* < 0.10), 0.38 (*P* < 0.01), 0.16 (*P* < 0.05) and 0.37 (*P* < 0.001) in sqrtMORT in ComB, ComC, ComG and OD, respectively, in comparison to those that had pigs from multiple origins. With respect to the herd age, batches belonging to ComE where farms were between 10 and 30 years old had a higher sqrtMORT by 0.18 compared to those newer than 10 years (*P* < 0.05). When batches from ComB were penned with less than 50 % of slatted floor, sqrtMORT decreased by 0.18 (*P* < 0.10) than those penned with more than 50 % of it. There was also a reduction in sqrtMORT by 0.23 (*P* < 0.05) and 0.25 (*P* < 0.001) when an automatic ventilation was available to batches belonging to ComB and OD respectively. There was an effect of the different methods of delivery of antibiotics treatment in ComB, showing an increase in sqrtMORT by 0.19 (*P* < 0.05) in those batches treated with antibiotics both in-feed and in-water compared to those only using antibiotics in-feed. A higher sqrtMORT at ComA (0.16; *P* < 0.05) was found in batches from farms that obtained drinking water from a river than from farms that had a public supply. Furthermore, the effect of the water source on sqrtMORT was modified by the IBW at ComE. Regarding the number of pigs placed, when compared to batches containing less than 800 pigs, those containing between 800 and 2000 pigs had an increase in sqrtMORT by 0.23 (*P* < 0.05) at ComB. In addition, there was also an increase by 0.36 (*P* < 0.05) and 0.29 (*P* < 0.01) in sqrtMORT in batches containing 800–2000 pigs and 0.63 (*P* < 0.05) and 0.33 (*P* < 0.001) in batches that contained more than 2000 pigs at ComF and OD respectively. Finally, for every 1 kg increase in IBW, sqrtMORT reduced 0.037 at ComD (*P* < 0.10) and 0.071 at ComE (*P* < 0.05).

**Table 4:** Parameter estimates (standard error in parenthesis) for the mortality rate (sqrtMORT) in each company studied^a^

Variable	Companies							
	A	B	C	D	E	F	G	OD^b^
Intercept	2.28 (0.46)^***^	1.92 (0.74)^*^	2.58 (0.67)^***^	2.23 (0.36)^***^	3.06 (0.64)^***^	1.48 (0.35)^***^	1.53 (0.42)^***^	2.25 (0.18)^***^
Autonomous community								
Cataluña	S	U	0.25 (0.13)^****^	U	U	U	S	S
Castilla y León	S	U	A	U	U	U	S	S
Valencia	S	U	−0.09 (0.18)	U	U	U	S	S
Other	S	U	A	U	U	U	S	S
Aragón	S	U	–	U	U	U	S	S
Trimester of placement								
Jan-Feb-Mar	A	−0.05 (0.12)	A	S	0.30 (0.13)^*^	A	S	−0.07 (0.06)
Apr-May-Jun	−0.04 (0.09)^****^	−0.45 (0.12)^***^	−0.25 (0.13)^*^	S	−0.14 (0.13)	−0.35 (0.16)^*^	S	−0.26 (0.05)^***^
Jul-Aug-Sep	A	−0.43 (0.14)^**^	A	S	−0.17 (0.12)	A	S	−0.28 (0.06)^***^
Oct-Nov-Dec	–	–	–	S	–	–	S	–
Circovirus vaccine								
No	0.57 (0.09)^***^	A	S	U	U	U	U	S
Yes	–	A	S	U	U	U	U	S
Number of pig origins								
One origin	S	−0.16 (0.11)^****^	−0.38 (0.15)^**^	U	U	U	−0.17 (0.08)^*^	−0.37 (0.05)^***^
More than one origin	S	–	–	U	U	U	–	–
Herd age								
<10 years	A	S	U	S	–	S	S	A
10 to 30 years	A	S	U	S	0.18 (0.09)^*^	S	S	A
>30 years	A	S	U	S	A	S	S	A
Percentage of slatted floor								
<50 % slatted	U	−0.18 (0.10)^****^	S	S	A	S	S	S
≥50 % slatted	U	–	S	S	A	S	S	S
Ventilation control system								
Manual	U	–	U	U	S	S	U	–
Automatic	U	−0.23 (0.12)^*^	U	U	S	S	U	−0.25 (0.05)^***^
Route utilized to perform treatments								
Feed	U	–	U	U	U	A	U	A
Feed + Water	U	0.19 (0.10)^*^	U	U	U	A	U	A
Feed + Water + Injection	U	A	U	U	U	A	U	A
Water source in the farm								
Well	−0.07 (0.13)	S	A	U	A	U	U	A
River	0.16 (0.08)^*^	S	A	U	A	U	U	A
Other	0.04 (0.14)	S	A	U	−1.92 (0.84)^*^	U	U	A
Public supply	–	S	A	U	–	U	U	A
Number of animals placed								
<800 pigs	S	–	S	S	S	–	S	–
800-2000 pigs	S	0.23 (0.10)^*^	S	S	S	0.36 (0.17)^*^	S	0.29 (0.06)^***^
>2000 pigs	S	A	S	S	S	0.63 (0.29)^*^	S	0.33 (0.07)^***^
Initial body weight	−0.03 (0.02)	−0.001 (0.03)	0.01 (0.03)	−0.04 (0.02)^****^	−0.07 (0.04)^*^	0.01 (0.02)	0.02 (0.02)	−0.001 (0.008)
Trimester * Circovirus vaccine								
Apr-May-Jun / No vaccinated	0.41 (0.20)^*^	U	U	U	U	U	U	U
/ Vaccinated	–	U	U	U	U	U	U	U
Oct-Nov-Dec / No vaccinated	–	U	U	U	U	U	U	U
/ Vaccinated	–	U	U	U	U	U	U	U
Initial BW * Water source								
Initial BW / Public wáter	U	U	U	U	–	U	U	U
/ Other	U	U	U	U	0.10 (0.05)^*^	U	U	U

S - variable was not selected to the final model; U - variable non-used in the model because it did not present variability; A - variable not available

(−) Reference level for a factor included in the multiple regression models

^*^ *P* < 0.05; ^**^ *P* < 0.01; ^***^ *P* < 0.001; ^****^ *P* < 0.10

^a^Data from 688 batches for companies A to F and 607 batches for OD. Variables not significant with respect to sqrtMORT in all companies studied and in OD were not described at the table

^b^OD - overall database. Data from ¨company G¨ was not included

## Discussion

Analyses of animal management and facility data showed that the variability between pig production companies is as important as within them. High homogeneity among farms within in the same company is a common practice in the Spanish pig companies and may be the same in countries were production is highly integrated. Oliveira et al. [7] evaluated factors affecting both mortality rate and feed intake of GF pigs in an integrated Spanish company and observed a narrow spectrum of husbandry and management practices among the different farms. Studies investigating factors that affect profitability in GF pigs were performed using batches from farms belonging to a unique company [6, 11] however, other studies [5, 8] looked at factors that affect profitability across companies using larger databases maintained by US Department of Agriculture (USDA). Both approaches can be used to study the variability within and among companies separately and provide different but complementary information.

Trimester of placement was an important factor responsible for variation in both FCR and sqrtMORT in four and five companies out of seven, respectively, as well as with OD. In most companies pigs placed in warm seasons had better performance. Maes et al. [6] also found higher mortality in pigs placed during the cold season compared to the warm season, and suggested that it could be due to respiratory diseases due to poorly ventilated buildings when trying to maintain indoor temperature. Thereby, its importance can vary among companies depending on facilities, management, or geographic location. All farms studied were located in northern Spain, and had similar outside temperature ranges, however there may have been differences in the ventilation of these farms depending on the age of the pig facility. On the other hand, environmental conditions were not recorded and trimester of placement may be correlated to other factors like quality and vailability of ingredients or meat demand. As a limitation of the study, not all companies reported the same range of categorical responses regarding trimester of placement. Three companies provided information of batches placed in the warm and cold season whereas others provided information of batches placed in all seasons. Although this difference should not cause important bias *a priori*, it has to be considered for future studies.

Variables such as breed of the pig-sire, sex, and sex segregation in batches were closely correlated among farms and the type of pig produced and they were pooled in a unique variable, BREGENSEG. Split-sex pens, use of entire males, and Pietrain-sire were normally used to produce “light” or “industrial” pigs whereas mixed-sex pens, which had White (Landrace, Large White, or their commercial crossings) and/or Duroc-sire pigs were preferred combinations to produce “heavy” pigs, although these combinations were also used in industrial production. In the present study six companies produced industrial pigs and one company produced heavy pigs. Only the industrial pig companies were included in the OD because of the specific production conditions of the heavy pig companies. According to Corrêa et al. [12] and Gispert et al. [13], the Pietrain boar is the most used breed of sire due to its high genetic potential converting feed into muscle tissue instead of fat tissue, improving feed efficiency. Differences observed in the companies presenting variability on this factor and in OD confirm and quantify this effect in the conditions of the current study.

Only two companies performed circovirus vaccination (ComA and ComC) in the GF phase. Circovirus disease (PCV2) was first described in Spain in 1997 and the infection is present in almost 100 % of Spanish pig farms [14]. However, a commercial vaccine against PCV2 was developed and introduced to the market in the last decade [15]. Currently, several companies produce this vaccine and it is used in different phases of production (sows, nursery piglets, and GF pigs). In the present study, companies that did not use this vaccination in GF phase may have done it in sows or piglets. Studies conducted by Segales et al. [16] and Jacela et al. [17] obtained a reduction in mortality and an improvement in feed efficiency when circovirus vaccine was performed in GF pigs. Similar results were obtained for ComA but not for ComC and OD. Differences in severity of the circovirus disease or in quality of the vaccine used by different companies could account for these differences.

Mixing piglets from different origins has been shown to increase disease transmission and decrease performance [18]. Maes et al. [6, 19] found an increase in mortality in batches that had pigs from multiple origins in a study using data from one Belgium pig company. In agreement, our data shows that batches using pigs from a single origin had better FCR and sqrtMORT both in the OD and in most of the companies presenting variability in this factor.

An analysis of the housing facilities showed that the percentage of slatted floor and type of ventilation system were significant factors only in ComB. Batches that had lower than 50 % of slatted floor or automatic ventilation control had lower sqrtMORT. In addition, automatic ventilation also led to a reduced sqrtMORT in OD. The optimal proportion of slated floor should be related to the density of pigs in the pen, since pigs use specific areas of the pen to feed, rest and defecate, where resting in a dry and solid floor is a priority [20]. In adittion, barns with fully slatted floor may have higher emissions of ammonia and other noxious gasses compared to those with partially slatted floors resulting in more respiratory problems and/or pulmonary lesions [21, 22]. The efficient removal of gases and moisture may depend also on the type of ventilation control system used. Choi et al. [23] observed higher profitability in nursery pigs housed in barns with automatic ventilation compared with those housed in manual ventilation. In contrast to our results, studies carried out by Losinger [5], Maes et al. [6], and Oliveira et al. [7] did not observe any influence of floor type or ventilation system on performance.

The type of feeder showed no effects in the individual companies in the current study, but it was an important facility factor in OD, where herds equipped with multi-space feeders had poorer FCR. Gonyou and Lou [24] and Myers et al. [25] concluded that single-space feeders with drinker may improve performance in GF pigs. On the other hand, Maes et al. [6] did not find any benefit using feeders with incorporated drinker.

Age of the barns, routes used to supply medication, and water source were factors not included in the OD model because there were missing values for a high number of batches. Thus, these factors were only studied at a single company level in order to obtain information about its effects. In three out of four companies, herds managed in facilities less than 10 years old had better FCR or lower sqrtMORT than herds that were managed in facilties that were more than 10 years old. There is a paucity of information regarding age of facilities on GF performance, however one study showed that age of facilities did not affect GF mortality rate [6]. Batches using only in-feed medication also had lower sqrtMORT. According Miller et al. [26], in-water and injection medication are more effective in sick pigs (for therapeutic purposes) whereas in-feed medication is associated with preventive medication. Thus, the use of medication only in feed may be more common in herds with excellent health. Results about water source were contradictory among companies and no clear conclusion can be drawn.

In agreement with our study Maes et al. [6] observed lower mortality in smaller batches. However Oliveira et al. [7] did not find an effect of batch size on mortality and feed intake. Our findings suggest that an all-in-all-out management system in small batches may improve health status.

Finally, IBW was included as a covariate in all the models to account for the large variability of IBW among companies unlike the FBW which was similar among companies. Forcing IBW was decided based on the experience of the authors with different data sets. Data from a single company may not have enough range of IBW to reach significance, however IBW was included to account for the corresponding variability. Lower IBW increased sqrtMORT and improved FCR in some companies, as also observed by Larriestra et al. [11] concerning mortality.

## Conclusions

In conclusion, the variability in management and facilities among Spanish companies was much higher than within them and some factors presented no variability within companies. Developing models for each company and for the overall data set provided complementary conclusions. Batches of grow-finishing pigs had better feed conversion ratio when: 1) were placed between April and September, 2) were originated from a Pietrain pig-sire, presence of males and segregated in pens, 3) came from a unique farm origin, 4) farms were newer than 10 years and 5) pigs had lower initial body weight. Batches of pigs had lower mortality rate when: 1) were placed between April and September, 2) pigs came from a unique farm origin, 3) the water was obtained from a well and/or public supply, 4) were raised in smaller farms (<800 pigs) and finally 5) pigs had higher initial body weight. Furthermore, due to the structure of the pig companies having common management practices and facilities in their farms, more research is necessary to investigate the factors affecting performance within and between companies, increasing the number of companies, herds and batches surveyed, as well as the number of factors studied in nutrition, welfare, biosecurity, and health.
